# Epithelial to mesenchymal transition is associated with rapamycin resistance

**DOI:** 10.18632/oncotarget.3669

**Published:** 2015-04-13

**Authors:** Ashley M. Holder, Argun Akcakanat, Farrell Adkins, Kurt Evans, Huiqin Chen, Caimiao Wei, Denai R. Milton, Yisheng Li, Kim-Anh Do, Filip Janku, Funda Meric-Bernstam

**Affiliations:** ^1^ Department of Surgical Oncology, The University of Texas MD Anderson Cancer Center, Houston, TX, USA; ^2^ Department of Surgery, Washington University in St. Louis, St. Louis, MO, USA; ^3^ Department of Investigational Cancer Therapeutics, The University of Texas MD Anderson Cancer Center, Houston, TX, USA; ^4^ Department of Colorectal Surgery, Cleveland Clinic Florida, Weston, FL, USA; ^5^ Department of Biostatistics, The University of Texas MD Anderson Cancer Center, Houston, TX, USA

**Keywords:** epithelial-to-mesenchymal transition (EMT), rapamycin, trametinib, biomarker, E-cadherin

## Abstract

Rapamycin analogues have antitumor efficacy in several tumor types, however few patients demonstrate tumor regression. Thus, there is a pressing need for markers of intrinsic response/resistance and rational combination therapies. We hypothesized that epithelial-to-mesenchymal transition (EMT) confers rapamycin resistance. We found that the epithelial marker E-cadherin protein is higher in rapamycin sensitive (RS) cells and mesenchymal breast cancer cell lines selected by transcriptional EMT signatures are less sensitive to rapamycin. MCF7 cells, transfected with constitutively active mutant Snail, had increased rapamycin resistance (RR) compared to cells transfected with wild-type Snail. Conversely, we transfected two RR mesenchymal cell lines—ACHN and MDA-MB-231—with miR-200b/c or ZEB1 siRNA to promote mesenchymal-to-epithelial transition. This induced E-cadherin expression in both cell lines, and ACHN demonstrated a significant increase in RS. Treatment of ACHN and MDA-MB-231 with trametinib modulated EMT in ACHN cells *in vitro*. Treatment of MDA-MB-231 and ACHN xenografts with trametinib in combination with rapamycin resulted in significant growth inhibition in both but without an apparent effect on EMT. Future studies are needed to determine whether EMT status is predictive of sensitivity to rapalogs and to determine whether combination therapy with EMT modulating agents can enhance antitumor effects of PI3K/mTOR inhibitors.

## INTRODUCTION

The PI3K/mTOR pathway performs essential functions for maintaining the malignant phenotype including controlling cell growth, metabolism, and autophagy [1, 2]. Rapamycin and its analogs are allosteric mTOR inhibitors that bind FKBP12 and mTOR, and predominantly inhibit mTORC1. The rapamycin analog temsirolimus is approved by the Food and Drug Administration for the treatment of advanced renal cell carcinoma and the rapamycin analog everolimus is FDA-approved for the treatment of pancreatic neuroendocrine tumors, renal cell carcinoma, sub-ependymal giant cell astrocytoma associated with tuberous sclerosis, and the treatment of hormone-receptor positive breast cancer (in combination with exemestane). However, rapalogs have shown objective responses in only a minority of patients. Mechanisms of intrinsic sensitivity and resistance to rapalogs remain largely unknown.

The epithelial to mesenchymal transition (EMT) is defined by the loss of intracellular links along with the gain of migratory and invasive abilities [3]. Plasticity exists within this process, allowing cells to transition from epithelial to mesenchymal and then resume an epithelial phenotype [4]. Snail and ZEB transcription factors are known EMT drivers, through inducing the crucial step of loss of cell polarity; their expression correlates with time to recurrence and survival in patients with breast carcinoma [3, 5]. Cell lines without E-cadherin expression or with mutations in E-cadherin have increased tumorigenicity and metastasis in mice [6–9]. Conversely, miR-200 has been shown to decrease the expression of ZEB transcription factors to maintain the epithelial phenotype [3, 10–14]. Forced expression of miR-200c restores the chemotherapeutic sensitivity of breast cancer cells [15], while loss of miR-200 correlates with increased vimentin expression and decreased E-cadherin expression in breast cancer cells [11, 13, 14].

Type 3 EMT is involved in cancer progression and metastasis, and thus it is a potential mechanism of attaining the malignant phenotype [4]. This phenotype is achieved through other common cancer signaling networks including MAPK, PI3K, and Smad [3]. MEK inhibitors, which target the MAPK pathway, have previously been shown to decrease vimentin expression and invasion in the triple negative breast cancer cell line MDA-MB-231 [16]. Trametinib (GSK1120212) is an orally bioavailable selective allosteric MEK1/MEK2 inhibitor that has been approved by the Food and Drug Administration (FDA) for treatment of *BRAF* V600 mutant melanoma in combination with dabrafenib and that is effective for inhibiting growth in triple negative breast cancer cell lines *in vitro* [17]. Histone deacetylase (HDAC) inhibitors, a class of antitumor agents, reverse EMT. Vorinostat, an FDA approved drug for the treatment of cutaneous T-cell lymphoma, is an inhibitor in this group that induces E-cadherin and inhibits vimentin expression [18].

Despite the cross-talk between EMT programming and the mTOR pathway, the relationship between rapamycin sensitivity in immortalized cancer cells lines and markers of EMT has not been previously investigated. We performed a functional proteomic screen with reverse phase protein array (RPPA) to determine biomarkers associated with sensitivity and resistance to rapamycin, and we found and association with EMT and rapamycin resistance. We hypothesized that the mesenchymal status of cancer cells imparts resistance to rapamycin. Thus, we proposed to modulate EMT in immortalized cancer cell lines and determine whether alterations in EMT biomarkers correlated with sensitivity to rapamycin both *in vitro* and in mouse xenografts.

## RESULTS

### Rapamycin sensitivity correlates with EMT status *in vitro*

**Figure 1:**
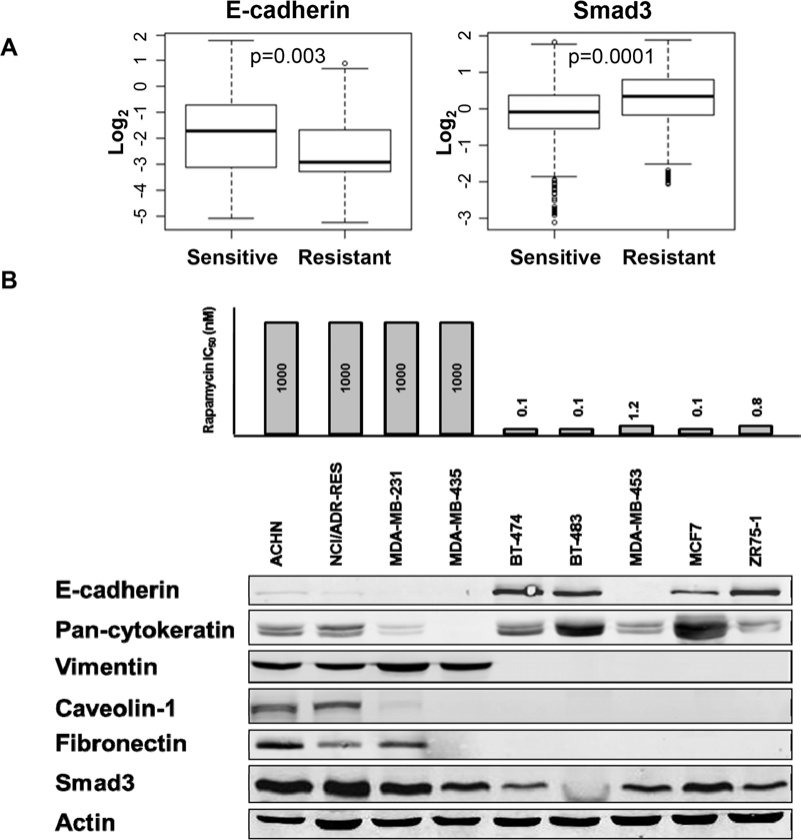
Rapamycin sensitive cell lines have increased E-cadherin expression compared to rapamycin resistant cell lines **A.** Forty three cell lines, with known rapamycin IC_50_ values, were treated with vehicle or increasing doses of rapamycin for 2, 24 or 72 hours in triplicates. Specific EMT markers were differentially expressed in RS compared to RR immortalized cell lines by RPPA. **B.** Western blotting assessed baseline expression of EMT markers in a panel of RS and RR cell lines.

To determine sensitivity to rapamycin in immortalized cancer cell lines, sulforhodamine B (SRB) assay was performed to classify cell lines as resistant or sensitive. Twelve cell lines with an IC_50_ of rapamycin greater than 100 nM were classified as resistant and 31 with IC_50_s less than 100 nM as sensitive [19]. Reverse phase protein arrays were used to compare the functional proteomics profiles. We assessed whether EMT markers are differentially expressed in rapamycin sensitive (RS) compared to resistant (RR) immortalized cell lines. RS cell lines demonstrated increased expression of epithelial marker E-cadherin while RR cell lines showed increased expression of mesenchymal marker Smad3 (Figure 1A). To validate these findings, western blotting for markers of EMT was performed in selected RR and RS cell lines. E-cadherin expression differentiated cell lines sensitive to rapamycin from those resistant to rapamycin, which expressed vimentin (Figure 1B). Thus, RS cell lines tended to display epithelial markers while RR cell lines exhibited mesenchymal markers.

**Figure 2:**
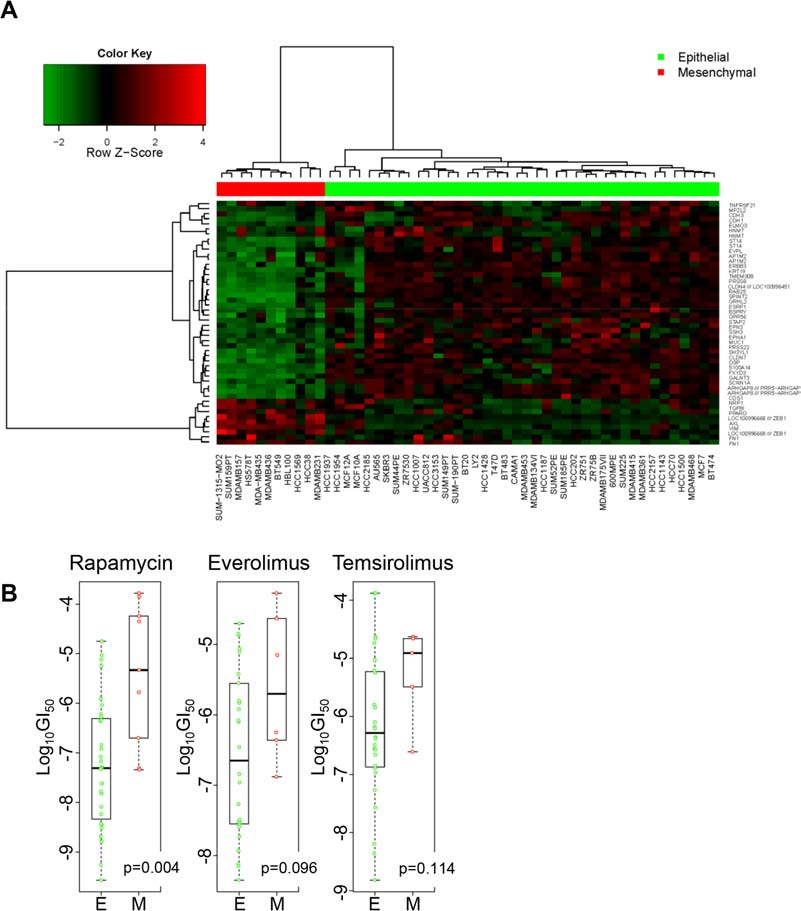
Sensitivity to rapamycin and analogs in epithelial and mesenchymal cell lines **A.** Breast cancer cell lines were grouped into epithelial (green bar) and mesenchymal (red bar) based on the Byers' EMT signature. Each row represents a gene, and each column a cell line. **B.** Wilcoxon rank sum test was performed to compare the Log_10_GI_50_ values between epithelial and mesenchymal cell lines.

Transcriptional signatures of EMT are being pursued as a predictor of response/sensitivity to selected therapies. We sought to determine whether transcriptional signature of EMT is associated with rapamycin sensitivity. Daemen et al. recently studied a panel of breast cancer cell lines and shared their gene expression profiles as well as sensitivity to experimental or approved therapeutic agents including rapamycin, as well a rapalogs everolimus and temsirolimus [20]. We classified these breast cancer cell lines as epithelial or mesenchymal based on their transcriptional profiles using genes within the EMT signature described by Byers et al. as a classifier [21]. Based on 2-way hierarchical clustering, 11 cell lines were clustered into the mesenchymal group (red bar) and 40 were clustered into the epithelial (green bar) group (Figure 2A). The ranks and the median of Log_10_GI_50_ of rapamycin were significantly lower in the epithelial group than the mesenchymal group (*p* = 0.004, Figure 2B). The median Log_10_GI_50_ of everolimus was lower in the epithelial group than the mesenchymal group (*p* = 0.096). The median Log_10_GI_50_ of temsirolimus was lower in the epithelial group than the mesenchymal group, but this difference did not achieve statistical significance. We also classified the breast cancer cell lines with 261 probe sets mapping to the 125 gene symbols of a EMT signature described Gröger et al. [22]. Based on 2-way hierarchical clustering, the classification of cell lines with this signature was very similar with 10 cell lines clustered as mesenchymal and 41 clustered as epithelial; HCC1569 was classified into the mesenchymal group by Byers' signature but epithelial group by Gröger's signature (Supplementary Figure 1). The median Log_10_GI_50_ of rapamycin was significantly lower in the epithelial group than that in the mesenchymal group with this classification as well (*p* = 0.004; Supplementary Figure 2).

### Induction of EMT alters Erk phosphorylation and decreases rapamycin sensitivity

**Figure 3:**
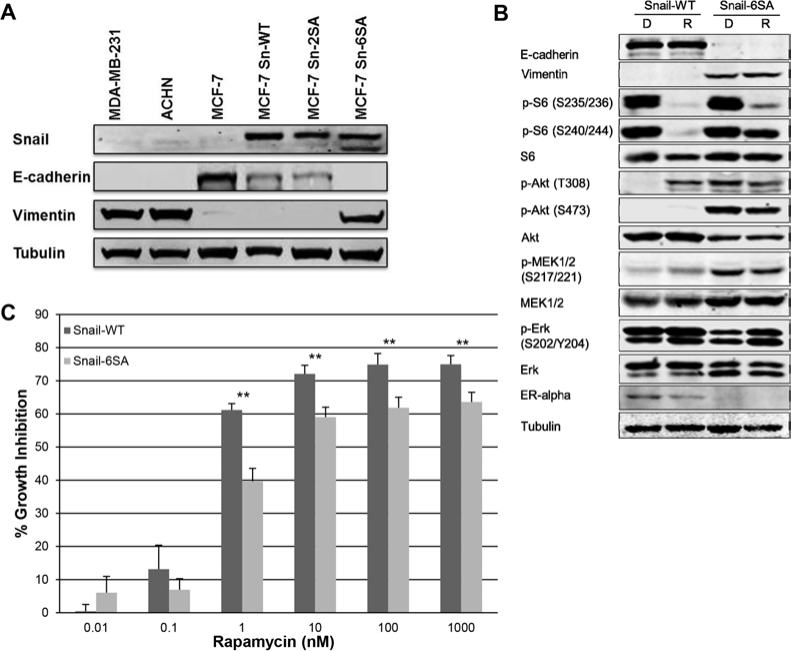
MCF7 Snail-6SA has increased Erk phosphorylation and decreased sensitivity to higher doses of rapamycin **A.** Snail, E-cadherin and vimentin expression was assessed in stably transfected MCF7 snail wild-type (Sn-WT) and MCF7 snail mutant (Sn-2SA and Sn-6SA) cell lines by western blotting. **B.** MCF7 snail wild-type (Snail-WT) and mutant (Snail-6SA) cell lines were treated with DMSO 0.1% or rapamycin 100 nM daily for 3 days. EMT, MAPK and mTOR pathway markers were assessed by western blotting. **C.** Rapamycin sensitivity in MCF7 snail wild-type (Snail-WT) or mutant (Snail-6SA) cell lines were assessed by SRB assay following 96-hour treatment with increasing doses of rapamycin. **p < 0.01. Experiments were performed three times in triplicates and a representative set of results is displayed.

To explore this correlation between rapamycin sensitivity and EMT further, we sought to modulate EMT and then study the effect on rapamycin sensitivity and mTOR signaling. To modulate EMT status in a stable manner, the epithelial breast carcinoma cell line, MCF7, had previously been transfected with a wild-type Snail (Snail-WT), and two mutant Snails (Snail-2SA and Snail-6SA) [23]. Snail-2SA is resistant to degradation by GSK-3β. Snail-6SA variant is also stable and a potent inducer of EMT in MCF7 cells. Western blotting for Snail, E-cadherin, and vimentin expression confirmed previous findings that MCF7 transfected with Snail-6SA mutant resulted in loss of E-cadherin expression and gain of vimentin expression (Figure 3A). The expression and phosphorylation of MAPK and Akt/mTOR pathway markers showed differences between MCF7 Snail-WT and Snail-6SA variants (Figure 3B). In Snail-6SA, rapamycin did not completely inhibit S6 phosphorylation. There was an increase in Akt phosphorylation but a decrease in total Akt expression. Total MEK expression was increased, which was accompanied by an increase in phospho-MEK (p-MEK). Contrary to this finding, Erk phosphorylation was decreased. In both Snail-WT and -6SA variants, rapamycin increased Erk phosphorylation, more significantly in Snail-6SA. To test the hypothesis that induction of EMT decreases sensitivity to rapamycin, MCF7 Snail-WT and MCF7 Snail-6SA were treated with varied doses of rapamycin. SRB assay exhibited statistically significant decrease in growth inhibition in MCF7 Snail-6SA at doses of rapamycin 0.01–1000 nM compared to MCF7 Snail-WT (Figure 3C). Induction of EMT in MCF7 resulted in increased baseline Erk phosphorylation, which was not regulated by rapamycin, and decreased sensitivity to rapamycin at higher doses, supporting our hypothesis that acquisition of mesenchymal markers and loss of epithelial markers imparts resistance to rapamycin.

### Modulation of EMT Increases Rapamycin Sensitivity in ACHN *In Vitro*

To test whether modulation of EMT to a more epithelial phenotype could increase rapamycin sensitivity in RR mesenchymal cell lines, we targeted the EMT program through transfection of miR-200b/c mimics and siRNA knockdown of ZEB1. We selected to attempt EMT modulation in two RR cell lines: the triple-negative breast carcinoma cell line, MDA-MB-231, and the renal cell carcinoma cell line, ACHN.

**Figure 4:**
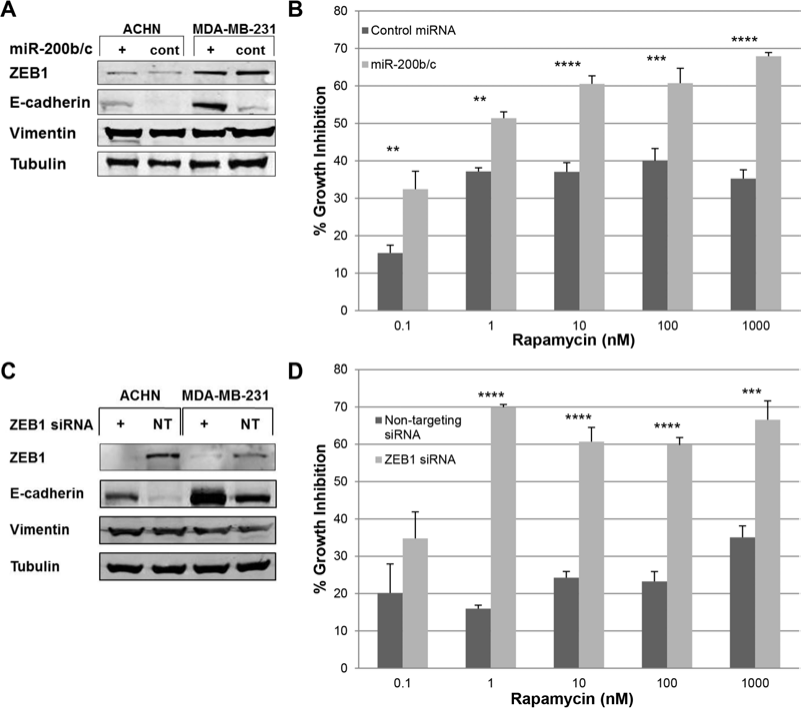
miR-200b/c transfection and ZEB1 siRNA knockdown modulated EMT in ACHN and MDA-MB-231 but increased sensitivity to rapamycin only in ACHN **A.** ACHN and MDA-MB-231 were transfected with miR-200b/c (+) and 72 hours later cells were harvested. EMT markers were assessed by western blotting. **B.** ACHN cell line was transfected with miR-200b/c (+) and 96 hours later growth inhibition was assessed by SRB assay following treatment with increasing doses of rapamycin. **p < 0.01; ***p < 0.001; ****p < 0.0001. Experiments were performed three times in triplicates and a representative set of results is displayed. **C.** ACHN and MDA-MB-231 were transfected with ZEB1 siRNA and 72 hours later cells were harvested. EMT markers were assessed by western blotting. **D.** Following ZEB1 siRNA knockdown, ACHN cells were treated with increasing doses of rapamycin for 96 hours. Cell growth inhibition was assessed by SRB assay. ***p < 0.001; ****p < 0.0001. Experiments were performed three times in triplicates and a representative set of results is displayed.

Both cell lines demonstrated increased E-cadherin expression with miR-200b/c transfection (Figure 4A). Despite increased E-cadherin expression in MDA-MB-231, there was no change in rapamycin sensitivity by SRB assay (data not shown). However, ACHN had increased sensitivity to rapamycin compared to control at all doses tested following miR-200b/c transfection, transforming from a RR to a RS cell line (Figure 4B).

Similarly, siRNA against ZEB1 was successful in abolishing ZEB1 expression in both ACHN and MDA-MB-231 compared to non-targeting siRNA, with concurrent increased expression of E-cadherin in both cell lines (Figure 4C). Similar to the miR-200b/c transfection, ZEB1 siRNA knockdown of MDA-MB-231 did not result in increased sensitivity to rapamycin compared to control (data not shown). However, ACHN demonstrated increased sensitivity to rapamycin 0.1–1000 nM following ZEB1 siRNA knockdown—once again converting from a RR to a RS cell line (Figure 4D).

Therefore, miR-200b/c transfection and ZEB1 siRNA knockdown modulated EMT in both ACHN and MDA-MB-231 but only increased the sensitivity of ACHN to rapamycin.

### HDAC inhibition modulates EMT and rapamycin sensitivity *in vitro*

**Figure 5:**
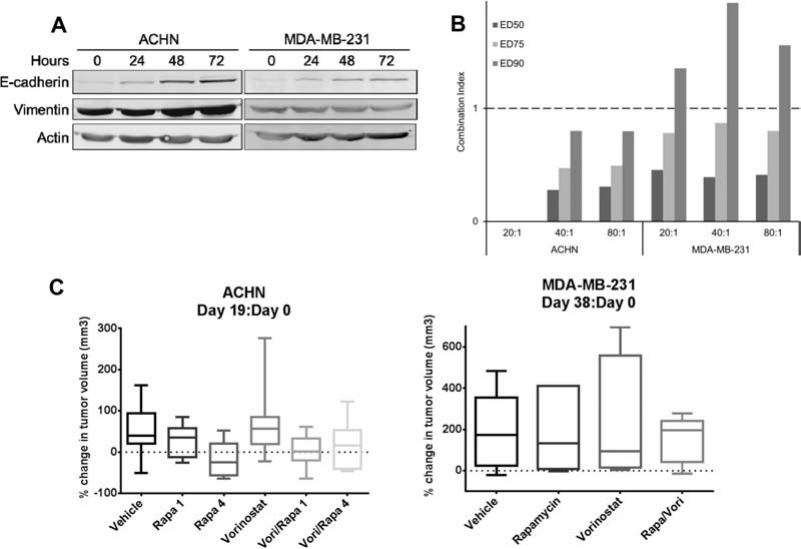
Vorinostat modulated EMT and showed synergistic effect with rapamycin *in vitro* but not *in vivo* **A.** ACHN and MDA-MB-231 cells were treated with vorinostat 5 μM for 24, 48 or 72 hours. Western blotting was conducted to assess EMT markers E-cadherin and vimentin. **B.** ACHN and MDA-MB-231 cell lines were treated with rapamycin and vorinostat for 96 hours. Rapamycin and vorinostat combination drug ratios were 20:1, 40:1, and 80:1. The effect on cell growth was assessed by SRB assay, and combination index (CI) values were calculated. The graph represents the CI of rapamycin and vorinostat combination at ED50, ED75, and ED90. Range of CI: <0.9, synergism; 0.9–1.1, nearly additive; >1.1, antagonism. **C.** ACHN xenografts were treated with vehicle, rapamycin 1 mg/kg (Rapa 1), rapamycin 4 mg/kg (Rapa 4), vorinostat 80 mg/kg, combination rapamycin 1 mg/kg and vorinostat 80 mg/kg (Vori/Rapa 1) or rapamycin 4 mg/kg and vorinostat 80 mg/kg (Vori/Rapa 4) for 19 days. MDA-MB-231 xenografts were treated with vehicle, rapamycin 1 mg/kg, vorinostat 80 mg/kg, or a combination of rapamycin and vorinostat (Rapa/Vori) at the same doses for 38 days.

As histone deacetylase (HDAC) inhibitors, have been reported to reverse EMT [18, 24], we determined if vorinostat reversed EMT in rapamycin resistant cell lines ACHN and MDA-MB-231. Treatment of these cell lines with vorinostat indeed increased E-cadherin levels (Figure 5A), demonstrating reversion of EMT. Increasing vorinostat dose did not increase E-cadherin expression (Supplementary Figure 3). The combination of rapamycin and vorinostat was strongly synergistic in ACHN at all concentrations and synergistic in MDA-MB-231 at lower concentrations (Figure 5B). To determine if vorinostat increased sensitivity to rapamycin *in vivo*, vorinostat alone or in combination with rapamycin was administered to MDA-MB-231 xenograft bearing mice for 38 days. Although the tumor volume in combination treatment group was slightly smaller than the single drug groups, there was no significant difference among the groups (Figure 5C). Notably, when expression of E-cadherin in MDA-MB-231 xenografts was analyzed by RPPA, there was no significant increase in E-Cadherin with treatment. To the contrary, there was a statistically significant decrease in E-Cadherin with vorinostat treatment (*p* = 0.0003); therefore vorinostat appeared to be unable to reverse EMT *in vivo* in this model.

In addition, vorinostat alone or in combination with two different doses of rapamycin was administered to ACHN bearing mice for 19 days. Compared to control, mean tumor volume decreased only in rapamycin 4 mg/kg group and was smaller in combination groups. There was no statistically significant difference among groups.

### MEK inhibition modulates EMT *in vitro* and the combination of MEK and mTOR inhibition enhances antitumor efficacy in ACHN models *in vivo*

**Figure 6:**
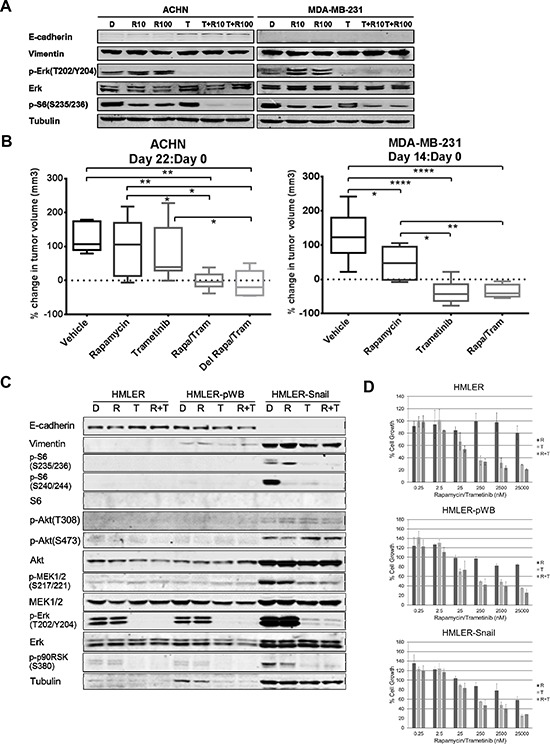
Trametinib modulated EMT in ACHN and increased sensitivity of ACHN to lower doses of rapamycin **A.** ACHN (left) and MDA-MB-231 (right) were treated daily with DMSO 0.1% (D), rapamycin 10 nM (R10), rapamycin 100 nM (R100), trametinib 10 μM (T), or in combination with each rapamycin dose (T+R10, T+R100) for 3 days and harvested for western blotting 24 hours later to assess EMT, MAPK and mTOR signaling. **B.** ACHN xenografts were treated with DMSO/Vehicle (Vehicle), rapamycin 1 mg/kg /vehicle (Rapamycin), DMSO/trametinib 0.3 mg/kg (Trametinib), rapamycin/trametinib (Rapa/Tram) at the same doses, or trametinib daily for three days then followed by rapamycin/trametinib (Del Rapa/Tram) at the same doses daily for a total of 14 days. *p < 0.05, **p < 0.01. MDA-MB-231 xenografts were treated with DMSO/Vehicle (Vehicle), rapamycin 1 mg/kg /vehicle (Rapamycin), DMSO/trametinib 0.3 mg/kg (Trametinib), or rapamycin/trametinib (Rapa/Tram) at the same doses daily for 14 days. *p < 0.05, **p < 0.01, ****p < 0.0001. **C.** HMLER, HMLER-pWB, and HMLER-Snail cell lines were treated daily with DMSO 0.1%, rapamycin 100 nM (R), trametinib 10 μM (T), or rapamycin and trametinib combination (R+T) with the same doses for 3 days. Cells were harvested for western blotting 24 hours after the last treatment and assessed for EMT, MAPK and mTOR signaling. **D.** HMLER (parenteral), HMLER-pWB (control vector), and HMLER-Snail were treated with various doses of rapamycin (R), trametinib (T) or their combination (R+T) for 4 days. Rapamycin:trametinib combination ratio was 1:1. Sensitivity to drugs was assessed by SRB assay. Results were normalized to DMSO control.

Previous studies have reported that MEK inhibition modulates EMT [16]. We thus tested whether the MEK inhibitor trametinib modulates EMT in ACHN and MDA-MB-231 cell lines. Trametinib inhibited MEK signaling without a significant decrease in vimentin or increase in E-cadherin in MDA-MB-231 cells (Figure 6A). In contrast, in ACHN cells, trametinib demonstrated an increase in E-cadherin levels, demonstrating some reversion of EMT. Also of interest is that in ACHN cells, rapamycin only partially abrogated ribosomal S6 phosphorylation. Although trametinib alone did not modulate ribosomal S6 phosphorylation, trametinib added to rapamycin completely inhibited S6 phosphorylation.

To determine if MEK inhibition increases sensitivity to rapamycin *in vivo*, effects of the combination of trametinib and rapamycin compared to single agent therapy was tested. Trametinib alone or in combination with rapamycin for 14 days caused tumor regression in MDA-MB-231 xenografts (Figure 6B), but the combination with rapamycin did not cause greater tumor regression than trametinib alone. Percent change in tumor volume from initiation of treatment (Day 0) was compared to that on the last day of treatment (Day 14). Percent change in tumor volume for all treatment groups were significantly less than control, but there was no difference in percent change in tumor volume between the mice treated with trametinib alone or with the combination of trametinib and rapamycin (Figure 6B). Notably western blotting for EMT markers did not demonstrate modulation of EMT with trametinib treatment despite inhibition of MAPK signaling (data not shown). Thus, trametinib caused tumor regression in MDA-MB-231 xenografts but did markedly modulate EMT nor did it further show antitumor efficacy in combination with rapamycin.

Given the differential response to EMT modulation with trametinib *in vitro* and the increased sensitivity to rapamycin observed with miR-200 transfection and ZEB1 knockdown, we next sought to evaluate the effect of trametinib on EMT markers and rapamycin sensitivity in an ACHN xenograft model. Similar to our results for MDA-MB-231 xenografts, trametinib caused tumor regression alone or in combination with rapamycin following 22-day treatment. However, the ACHN xenografts demonstrated greater tumor regression with trametinib in combination with rapamycin, whether trametinib was given prior to rapamycin treatment or simultaneously (Figure 6B). The percent change in tumor volume from Day 0 to Day 22 of treatment demonstrated greater tumor regression with the combination of trametinib and rapamycin and a trend towards greater tumor regression with the pre-treatment trametinib combination group compared to simultaneous treatment (Figure 6B).

As trametinib not only modulates EMT, but also inhibits MAPK signaling, we further investigated the role of trametinib on cell growth on a MAPK-activated model. HMLER is an immortalized human mammary epithelial cell line (HMLE) transformed with V12H-Ras oncogene and rendered oncogenic [25, 26]. HMLER and HMLER-pWB (control vector) both had baseline activation of MAPK signaling. MAPK pathway signaling was further activated in HMLER-Snail and blocked with trametinib alone or in combination with rapamycin (Figure 6C). Trametinib alone inhibited both pathways but there was only a slight decrease in vimentin expression, not suggesting reverting EMT. Rapamycin was not able to block S6 phosphorylation completely. Of the markers examined, combination treatment did not result in more inhibition compared to trametinib alone. Tubulin was also increased in HMLER-Snail cell line; consistent with previous reports that reorganization of cytoskeleton causes increase in actin and tubulin expression [27–29]. A similar increase was also observed in MEK, Erk, and Akt. We have treated HMLER (parenteral), HMLER-pWB (control vector), and HMLER-Snail cells with increasing doses rapamycin and trametinib alone and in combination (Figure 6D). Trametinib alone or in combination with rapamycin inhibited cell growth in all three panels at doses of 25 nM or higher. However, in HMLER-Snail model, reverting EMT did not provide more growth inhibition to combination with rapamycin compared to trametinib alone.

## DISCUSSION

We found an association with EMT and rapamycin resistance on a functional proteomic screen. Further, we demonstrated that mesenchymal cell lines selected by an EMT signature are more resistant to rapamycin. We thus hypothesized that the mesenchymal status of cancer cells, exhibited through absence of E-cadherin expression and presence of vimentin expression, correlated with resistance to rapamycin both *in vitro* and in mouse xenografts. However, some, but not all mechanisms of EMT modulation resulted in increased sensitivity to rapamycin in cancer cell lines *in vitro* and *in vivo*.

The rapamycin analog temsirolimus is approved by the Food and Drug Administration for the treatment of advanced renal cell carcinoma, and the rapamycin analog everolimus is FDA-approved for the treatment of pancreatic neuroendocrine tumors, renal cell carcinoma, sub-ependymal giant cell astrocytoma associated with tuberous sclerosis, and hormone-receptor positive breast cancer (in combination with exemestane). There has been intensive study on mechanism of action and mechanisms of intrinsic sensitivity and resistance to rapamycin, but largely, biomarkers predictive for response to rapalogs have not been identified. Although both somatic TSC1 mutation [30] and mTOR mutations [31] have been reported in exceptional responders, these alterations are relatively rare in tumor types such as hormone–receptor positive breast cancer and neuroendocrine tumors, where rapalogs are commonly used. In preclinical studies PIK3CA/PTEN mutations were associated with rapalog sensitivity [19], but to date PIK3CA mutations have not been confirmed to predict response to rapalogs in clinical trials [32]. It is also worth noting that rapalogs are associated with toxicities, including stomatitis of mild- to moderate severity including, pneumonitis, and hyperglycemia [33]. Although these side effects are usually manageable, they highlight the need to identify biomarkers of response to spare patients who will not benefit from these agents these side effects.

There were differences in expression and phosphorylation of MAPK and Akt/mTOR pathway markers in MCF7 Snail-WT and Snail-6SA cell lines. Rapamycin was reported to activate MAPK pathway through S6K-PI3K-Ras feedback loop in various models [34, 35]. MAPK activation accompanied with incomplete inhibition of S6 phosphorylation and Akt activation indicated resistance to rapamycin, and provided rationale for a combination of MAPK and mTOR inhibitors in treatment of cancer.

EMT has been associated with chemoresistance to several chemotherapeutic agents including paclitaxel [36], oxaliplatin [37], gemcitabine [38], 5-fluororacil [39] as well as targeted therapies such as tamoxifen [40], erlotinib and PI3K/Akt inhibitors [21]. RNA-based multiplex predictors such as Oncotype Dx Recurrence Score and MammaPrint have been effectively transitioned to clinical use; thus, it would be important to determine whether a RNA- or protein-based predictor can indeed have predictive utility. In our study, cell lines that were classified as mesenchymal based on two EMT signatures were less sensitive to rapamycin; although sensitivity to everolimus and temsirolimus was less, this difference did not reach statistical significance. It is worthy of note that in contrast to our *in vitro* findings, metaplastic breast cancer, a mesenchymal tumor type, has previously been reported to be especially responsive to a temsirolimus containing therapy regimen; 42% objective response rate was reported for temsirolimus, bevacizumab and liposomal doxorubicin [41]. Whether this sensitivity is attributable to sensitivity to temsirolimus or bevacizumab and liposomal doxorubicin remains unclear. Thus, further study is needed to determine whether EMT correlates with clinical resistance (early progression or lack of clinical benefit) to rapalogs as single agent as well in combination therapy.

A variety of preclinical approaches has been proposed to reverse EMT. In our experiments, in each mechanism of EMT modulation *in vitro*, ACHN demonstrated increased E-cadherin expression and increased sensitivity to rapamycin. In addition, vorinostat and trametinib treatments induced E-cadherin expression. However, *in vivo* neither of the drugs increased E-cadherin expression, and rapamycin and vorinostat combination had no effect on tumor growth. Thus, *in vitro* findings were not capitulated with *in vivo* results. Notably our results differ than those reported in epidermoid squamous cell carcinoma xenografts, where vorinostat both reversed EMT and inhibited tumor growth [24]. In addition to differences in cell lines used to establish the xenografts, at least one difference may be that in our model we started treatment after tumors were established (mean volume ± SEM, 118 ± 15 mm^3^) whereas the other model introduced an early treatment protocol that started on day 3 [24]. However, we feel treatment of established tumors better models treatment of patients with advanced disease in early phase clinical trials.

We observed an increase in E-cadherin expression in MDA-MB-231 by miR200b/c transfection and ZEB1 knockdown, however, there was no growth inhibition. MDA-MB-231 has BRAF and RAS mutations that may render MDA-MB-231 resistant to rapamycin regardless of EMT status. This was further supported by our findings with HMLER cell line, and in the presence of RAS mutation, reverting EMT by trametinib treatment did not induce sensitivity to rapamycin. In ACHN cell line, E-cadherin was increased after miR200b/c or ZEB1 transfections and trametinib treatment, which may have been adequate to alter EMT and its crosstalk with PI3K/mTOR signaling, ultimately to result in increased sensitivity to rapamycin. Simultaneous targeting of Ras/Raf/MEK/Erk and PI3K/Akt/mTOR pathways has already been proposed as an approach to overcome resistance to Raf-i or MEK inhibitors [42, 43]. In both MDA-MB-231 and ACHN cell lines, trametinib alone or in combination with rapamycin was better than rapamycin alone, but we were unable to demonstrate reversion of EMT in these models. In HMLER cell lines, trametinib alone or in combination with rapamycin inhibited growth more than rapamycin alone. There was a decrease in vimentin expression but we did not capture an increase in E-cadherin expression and we were not able to demonstrate reversion of EMT clearly. We did not capture an increase in p-S6K (data not shown), which is upstream S6. Although p-S6 was increased in HMLER-Snail cell line, probably PI3K pathway was not activated but rather MEK/Erk/p90RSK axis phosphorylated S6. Trametinib alone was able to inhibit activity of both MAPK and PI3K/mTOR pathways. Our finding raises the possibility that trametinib's synergy with rapamycin may be through mechanisms independent of MET.

Future studies are needed to determine optimal agents for reversion of EMT modulation and to determine the effect of combinations of these agents with PI3K/Akt/mTOR inhibitors.

## MATERIALS AND METHODS

### Cell lines and culture

ACHN, BT-474, BT-483, MCF7, MDA-MB-231, MDA-MB-453, and ZR75–1 cell lines were obtained from the American Type Culture Collection. MDA-MB-435 and NCI/ADR-RES cells were obtained from the National Cancer Institute. Cell lines were passaged for less than six months following resuscitation, and thus were not tested for characterization. ATCC utilizes Short Tandem Repeat (STR) profiling to verify cell line identity. Cells were cultured in DMEM/F12 supplemented with 10% fetal bovine serum at 37°C and humidified 5% CO_2_. MCF7 cell lines containing Snail-WT, Snail-2SA or Snail-6SA were created as previously described [23] and grown in DMEM/F12 supplemented with 10% fetal bovine serum and G418 400 μg/ml. HMLER (parenteral), HMLER-pWB (control vector) and HMLER-Snail cells were cultured in DMEM/F12:MEGM (with BPE supplement) (1:1) media supplemented with hEGF 5 ng/ml, hydrocortisone 0.25 μg/ml, insulin 2.5 μg/ml, and blasticidin 4 μg/ml.

### Reagents

Rapamycin and vorinostat were purchased from LC Laboratories, Inc. Trametinib was purchased from Selleck Chemicals. For *in vivo* experiments, 0.5% hydroxypropylmethylcellulose (Sigma) and 0.2% Tween-80 (Sigma) in distilled water (pH 8.0) was used as oral gavage vehicle as previously described [17]. DMSO (vehicle for rapamycin for *in vitro* and *in vivo* experiments), polyethylene glycol (vehicle for vorinostat for *in vivo* experiments), and G418 were purchased from Sigma.

### Western blotting

Cells were washed with cold PBS and lysed in 100 mM Tris-HCl (pH 6.8), 4% SDS, and 20% glycerol, and then protein were separated by SDS-PAGE. The protein was transferred to a 0.2 μm nitrocellulose membrane (Bio-Rad Laboratories). Membranes were blocked with 0.1% casein in TBS. Immunoblotting was performed with the following antibodies: caveolin-1, pan-cytokeratin, E-cadherin, Erk 1/2, p-Erk 1/2 (Thr202/Tyr204), MEK1/2, p-MEK1/2 (S217/221), p-S6 (Ser240/244), p-S6 (Ser235/236), S6, p-Akt T308, p-Akt S473, Akt, p90RSK S380, α-tubulin (Cell Signaling Technology), fibronectin, ERα, smad3 (Epitomics), Snail, vimentin (Abcam), actin (Sigma), and ZEB1 (Bethyl). The immunoblots were visualized using the Odyssey IR imaging system and software (Li-Cor Biosciences).

### Reverse phase protein arrays

Reverse phase protein array (RPPA) was performed at the MD Anderson Cancer Center Functional Proteomics RPPA Core Facility as described previously [44–46], and specifically represents cells and samples with two biological replicates prepared as previously described [19]. Protein levels were presented as log_2_ of mean expression values.

### Small interfering rna

The silencing of ZEB1 with small interfering RNA (siRNA) was performed using DharmaFECT 1 transfection reagent and siGENOME SMARTpool Human ZEB1 siRNA (GAACCACCCUUGAAAGUGA, GAAGCAGGAUGUACAGUAA, AAACUGAACCUGU GGAUUA, GAUAGCACUUGUCUUCUGU) or siGENOME Non-targeting siRNA Pool #2 (Dharmacon). Cells were harvested after 72 hours and lysates obtained for western blotting or trypsinized and plated for growth assay experiments.

### Microrna

The transient transfection of miR-200b (CCAGCUCGGGCAGCCGUGGCCAUCUUACUGGG CAGCAUUGGAUGGAGUCAGGUCUCUAAUACUG CCUGGUAAUGAUGACGGCGGAGCCCUGCACG) and miR-200c (CCCUCGUCUUACCCAGCAGUGUUU GGGUGCGGUUGGGAGUCUCUAAUACUGCCGGG UAAUGAUGGAGG) in a 50:50 ratio or Negative Control miRNA #1 was performed using reverse transfection method and siPORT *NeoFX* transfection reagent (Ambion). Cells were harvested after 72 hours and lysates obtained for western blotting or trypsinized and plated for growth assay experiments.

### Growth assays

For rapamycin antiproliferative activity, cells were plated in triplicate in 96-well plates at densities of 500 to 5, 000 cells per well depending on growth characteristics of each cell line. Cell growth was measured at 5 days (baseline rapamycin sensitivity classification) and 4 days (miR-200 transfection and ZEB1 knockdown) using sulforhodamine B (SRB) assay as previously described [47]. The median inhibitory concentration (IC_50_) and combination index (CI) were determined from dose-response curves as previously described [48]. Cells were categorized as rapamycin sensitive (RS) or rapamycin resistant (RR) based on IC_50_ cutoff value of 100 nM.

### *In vivo* studies

All animal studies were conducted according to the guidelines of the American Association of Laboratory Animal Care under a protocol approved by the MD Anderson Animal Care and Use Committee. ACHN (6.67 × 10^6^) and MDA-MB-231 (1 × 10^7^) cells, mixed with Matrigel (BD Biosciences), were inoculated into the mammary fat pads of six-week-old female athymic nude (nu/nu) mice (Department of Experimental Radiation Oncology, MD Anderson). In trametinib-rapamycin study, after tumors formed, MDA-MB-231 xenografts were randomized into 4 groups (DMSO intraperitoneal injection (IP) weekly/oral gavage vehicle daily, DMSO IP weekly/trametinib 0.3 mg/kg body weight oral gavage daily, rapamycin 1 mg/kg IP weekly/oral gavage vehicle daily, or rapamycin 1 mg/kg IP weekly/trametinib 0.3 mg/kg body weight oral gavage daily, *n* = 7–8). After tumors formed, ACHN xenografts were randomized into 5 groups (DMSO IP weekly/oral gavage vehicle daily, DMSO IP weekly/trametinib 0.3 mg/kg body weight oral gavage daily, rapamycin 1 mg/kg IP weekly/oral gavage vehicle daily, rapamycin 1 mg/kg IP weekly/trametinib 0.3 mg/kg body weight oral gavage daily, or trametinib 0.3 mg/kg body weight oral gavage daily for three days followed by simultaneous rapamycin 1 mg/kg IP weekly/trametinib 0.3 mg/kg body weight oral gavage daily *n* = 8). In vorinostat-rapamycin study, after tumors formed, MDA-MB-231 xenografts were randomized into 4 groups (vehicle 10% DMSO-PEG:water IP three times per week, rapamycin 1 mg/kg IP weekly, vorinostat 80 mg/kg IP three times per week, rapamycin 1 mg/kg IP weekly/vorinostat 80 mg/kg IP three times per week, *n* = 5). After tumors formed, ACHN xenografts were randomized into 6 groups (vehicle 10% DMSO-PEG:water IP three times per week, rapamycin 1 mg/kg IP weekly, rapamycin 4 mg/kg IP weekly, vorinostat 80 mg/kg IP three times per week, rapamycin 1 mg/kg IP weekly/vorinostat 80 mg/kg IP three times per week, rapamycin 4 mg/kg IP weekly/vorinostat 80 mg/kg IP three times per week, *n* = 8). The tumor growth was followed by caliper measurements and tumor volumes were calculated as previously described [49]. Mice were euthanized 24 hours after the last treatment, and half of each tumor was snap-frozen while the other half was fixed in formalin and embedded in paraffin.

### Statistical analysis

The cell line RPPA slide data was analyzed for differences in expression between RS and RR cell lines using linear mixed effects model with fixed effect of sensitivity and time point, and random effect of cell line. To account for multiple testing, we estimated the false discovery rates (FDR) of the F-tests of the cell line sensitivity effect using beta-uniform mixture model. All results shown in bar graphs and line graphs are presented as means ± SE. Growth inhibition (SRB data) of MCF7 Snail-WT v. MCF7 Snail-6SA following rapamycin treatment was compared with a Student's *t*-test. Growth inhibition with rapamycin (SRB data) for miR-200 transfection and ZEB1 knockdown was compared to respective control using 2-Way ANOVA for treatment v. rapamycin/DMSO followed by Bonferroni multiple comparison tests. For trametinib-rapamycin and vorinostat-rapamycin *in vivo* experiments, tumor volume at the last day and percent change in tumor volume from day 0 to the last day were both compared among all treatment groups by 1-way ANOVA followed by Tukey multiple comparison test or Kruskal Wallis test followed by Dunn's multiple comparison test. All statistical tests used a significance level of 5%.

For the analysis of association between breast cancer cell line sensitivity and EMT signature, we defined the cell line sensitivity by using the average of GI_50_ values on Log_10_ scale of all cell lines available in GI_50_ data from Daemen et al. for rapamycin, everolimus or temsirolimus respectively [20]. We extracted the gene expression information from data from Daemen et al. that matches Byers and Gröger EMT signature genes for the cell lines with GI_50_ and sensitivity information for the compounds of interest. For the Byers EMT signature, probe set ID was used as the identifier. There are 48 probe sets in Daemens's Affymetrix U133A data mapping to Byers' 97 probe sets signature from Affymetrix U133plus2 data. For Gröger's signature, gene symbol was used as identifier and multiple probe sets for the same gene symbols were extracted when available. We conducted similar analysis for Byers' and Gröger's EMT signatures on the probe set level. Two-way hierarchical clustering was conducted to cluster Affymetrix probe sets corresponding to the EMT signature genes using Pearson correlation distance metric and cluster cell lines using Euclidean distance metric with the Ward's linkage rule. The cell lines were grouped into epithelial (green bar) and mesenchymal (red bar) groups at the first major branching of the dendrogram. Wilcoxon rank sum tests were used to compare the Log_10_GI_50_ values between epithelial and mesenchymal cell lines.

## SUPPLEMENTARY FIGURES


