# Supplementary material for: Epithelial to mesenchymal transition is associated with rapamycin resistance
Source: Oncotarget. 2015 Apr 13;6(23):19500–13. doi: 10.18632/oncotarget.3669 (PMC4637301; doi:10.18632/oncotarget.3669)
Supplement: Supplementary file 1 [file oncotarget-06-19500-s001.pdf]

SUPPLEMENTARY FIGURES

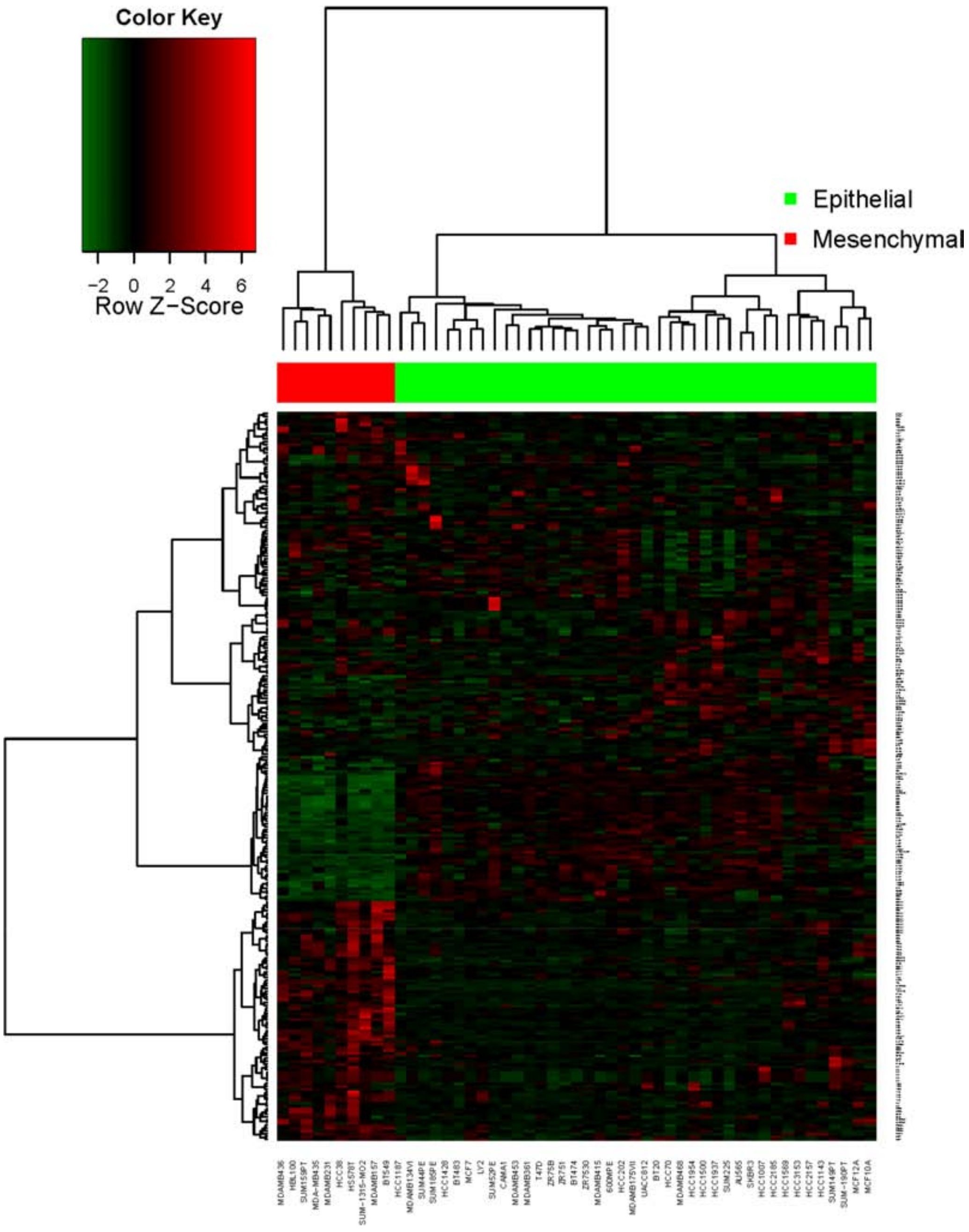

Supplementary Figure S1: Breast cancer cell lines were grouped into epithelial (green bar) and mesenchymal (red bar) based on the Gröger's EMT signature. Each row represents a gene, and each column, a cell line.

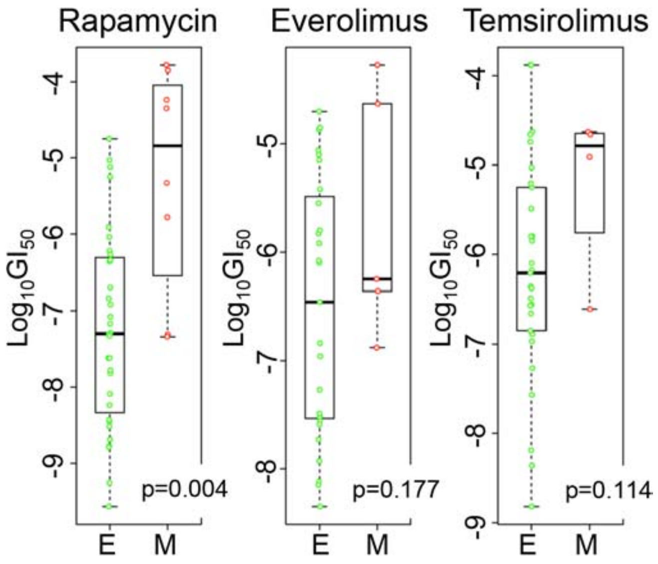

Supplementary Figure S2: Wilcoxon rank sum test was performed to compare the $Log_{10}GI_{50}$ values between epithelial and mesenchymal cell lines by using Gröger's EMT signature.

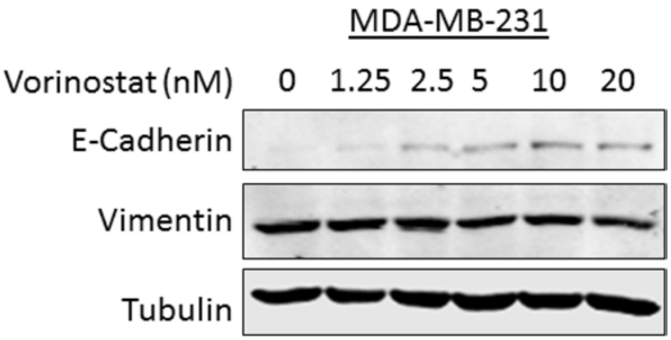

Supplementary Figure S3: MDA-MB-231 cells were treated with increasing doses of vorinostat. EMT markers were assessed by immunoblotting.
